# Photovoltaic Performance of a Nanowire/Quantum Dot Hybrid Nanostructure Array Solar Cell

**DOI:** 10.1186/s11671-018-2478-5

**Published:** 2018-02-23

**Authors:** Yao Wu, Xin Yan, Xia Zhang, Xiaomin Ren

**Affiliations:** State Key Laboratory of Information Photonics and Optical Communications, Beijing University of Posts and Telecommunications, Beijing, 100876 China

**Keywords:** Nanowire array, Quantum dot, Photovoltaic, Nanophotonics and photonic crystals

## Abstract

An innovative solar cell based on a nanowire/quantum dot hybrid nanostructure array is designed and analyzed. By growing multilayer InAs quantum dots on the sidewalls of GaAs nanowires, not only the absorption spectrum of GaAs nanowires is extended by quantum dots but also the light absorption of quantum dots is dramatically enhanced due to the light-trapping effect of the nanowire array. By incorporating five layers of InAs quantum dots into a 500-nm high-GaAs nanowire array, the power conversion efficiency enhancement induced by the quantum dots is six times higher than the power conversion efficiency enhancement in thin-film solar cells which contain the same amount of quantum dots, indicating that the nanowire array structure can benefit the photovoltaic performance of quantum dot solar cells.

## Background

The incorporation of quantum dots (QDs) into solar cells has been proposed as a promising way to enhance the device conversion efficiency [1, 2]. Insertion of QDs into the active region of a solar cell allows one to engineer the effective bandgap of the material and extend the absorption spectrum [3–6]. This can be used to enhance the photocurrent of a homogeneous solar cell [7–9] or to form an isolated intermediate band within the bandgap of the host material to absorb photons with energy below host material energy gap [10–13]. However, to surpass the efficiency of conventional devices, the absorption enhancement caused by QDs must be improved significantly. This may be achieved by increasing the number of QDs, by enhancing the optical absorption, or a combination of both [14]. In recent years, an attractive structure has been fabricated by growing Stranski-Krastanov (S-K) QDs on the sidewalls of nanowires (NWs), which offers an innovative approach to combine the advantage of the two kinds of nanostructures [15–19]. Multilayer QDs can be grown on the sidewalls of NWs, which substantially increase the number of QDs, while the vertically aligned NW array can dramatically enhance the absorption of QDs due to the excellent light-trapping ability [20–24]. Thus, the photocurrent contributed by QDs in the NW/QD hybrid nanostructure array is expected to be larger than that in thin-film QD structures. Moreover, the NW/QD hybrid structure can be fabricated on low-cost silicon substrates, which makes it promising for low-cost, high-efficiency solar cells [25]. Although the fabrication and optical properties of NW/QD hybrid nanostructures have been widely reported, the performance of solar cells based on the hybrid structures has not been investigated yet.

In this paper, a coupled optoelectronic simulation is presented to investigate the photovoltaic performance of a GaAs/InAs NW/QD hybrid solar cell. The considered structure consists of a vertically aligned NW array with each NW containing five layers of QDs arranged perpendicular to the NW growth axis. Both the QDs and the wetting layers (WLs) contribute to sub-bandgap photon absorption, extending the absorption spectrum to 950 nm. Each NW consists of a radial pin junction with all of the QD layers located in the intrinsic region. At first, a comparison in light absorption spectra between the NW arrays with and without QDs is made by using three-dimensional finite-difference time-domain (3D-FDTD) simulations. The absorption spectra of their thin-film counterparts are calculated as well. Then, the photogeneration profiles are incorporated into the electrical simulations to calculate the current density versus voltage (*I*-*V*) characteristics. The results show that, in both NW array and thin-film solar cells, incorporation of QDs can enhance the short-circuit current (*J*_sc_) by increasing light absorption; however, a degeneration of open-circuit voltage (*V*_oc_) occurs at the same time. The overall power conversion efficiency enhancement induced by the quantum dots in NW array solar cells is six times higher than the efficiency enhancement in thin-film solar cells which contain the same amount of quantum dots, indicating that the NW array structure can benefit the photovoltaic performance of quantum dot solar cells.

## Methods

In our previous study [15], the fabrication of the NW/QD hybrid structures was realized by using a Thomas Swan Close Coupled Showerhead (CCS) metal organic chemical vapor deposition (MOCVD) system. Trimethylgallium (TMGa), trimethylindium (TMIn), and arsine (AsH_3_) were used as precursors. The carrier was hydrogen. An Au-coated GaAs substrate was loaded into the MOCVD reactor and annealed under AsH_3_ ambient to form Au-Ga alloy particles as catalyst. The GaAs NWs were grown in the first place, and then the first shell of InAs QDs was deposited by switching off TMGa and raising the temperature. After the growth of the InAs QD layer, the GaAs spacer shell was radially grown on the InAs QDs. The multilayers of QD structures were realized by repeating the combination of InAs QDs and GaAs spacer shell for certain times.

**Fig. 1:**
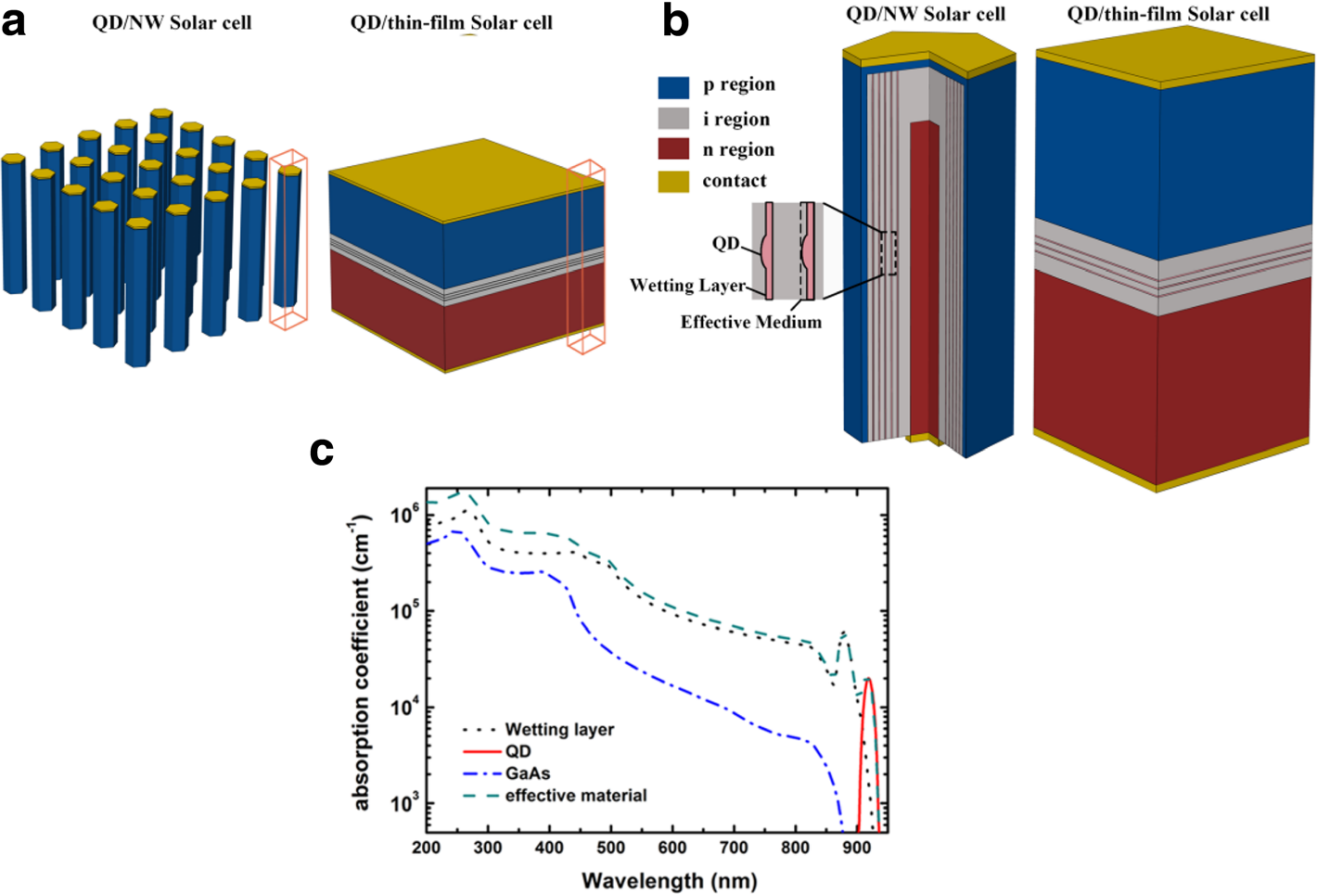
**a** The schematic drawings of the NW/QD hybrid solar cell and its thin-film counterpart. **b** The detailed structures of the units marked with wireframes in **a**. **c** Absorption coefficient of the effective medium. The volume fractions of QDs, WL, and GaAs in the effective medium are 0.002882996, 0.649123, and 0.347994, respectively

The schematic of the NW/QD hybrid solar cell is illustrated in Fig. 1a. The device consists of periodic GaAs/InAs NW/QD hybrid structures. Each NW contains a radial pin junction with five layers of QDs arranged perpendicular to the NW growth axis in the intrinsic region, as shown in Fig. 1b. The doping concentration of the p-type shell and n-type core is 3 × 10^18^ and 1 × 10^18^ cm^−3^, respectively. The QD layers are modeled by treating InAs QDs, WL, and GaAs material surrounding QDs as an effective medium. The thickness of each effective medium is 2 nm.

For optical simulation, the wavelength-dependent complex refractive index of the effective medium is calculated by a volume-weighted superposition of the QDs, WL, and GaAs material as described in [26], which is expressed by Eq. (1).

1 $$ {\alpha}_{\mathrm{eff}}={F}_{\mathrm{QD}}{\alpha}_{\mathrm{QD}}+{F}_{\mathrm{WL}}{\alpha}_{\mathrm{WL}}+{F}_{\mathrm{GaAs}}{\alpha}_{\mathrm{GaAs}} $$

where *F*_QD_, *F*_WL_, and *F*_GaAs_ are the volume fractions of QD, WL, and GaAs materials in the effective medium, respectively. The absorption coefficient of InAs QDs and WLs is obtained from [26], with the same QD size and density. Other material parameters are obtained from [27]. The absorption coefficient used in this work is presented in Fig. 1c. Two peaks are observed below GaAs bandgap, with one centered at a wavelength of 876 nm and the other centered at 916 nm, which are attributed to the QD layers. The thin-film solar cell containing QD layers is also simulated for comparison. Thickness of the thin-film solar cell is set to be equal to the NW length, and the total volume of QD layers and the thickness of the intrinsic layer in the thin-film solar cells are set to be the same with those in the NW/QD hybrid solar cells. The absorption properties of the solar cells are calculated by the FDTD Solutions software package (Lumerical Solutions, Inc.). By placing periodic boundary conditions, the simulations can be carried out in a single unit cell to model the periodic array structure. The AM1.5G spectrum is divided into 87 discrete wavelength intervals, from 300 to 950 nm. The transverse electric (TE) and transverse magnetic (TM) mode contributions are superimposed to model the corresponding unpolarized feature of sunlight. The total optical generation under AM1.5G illumination can be modeled by superimposing the spectrally resolved single-wavelength photogeneration rates.

**Fig. 2:**
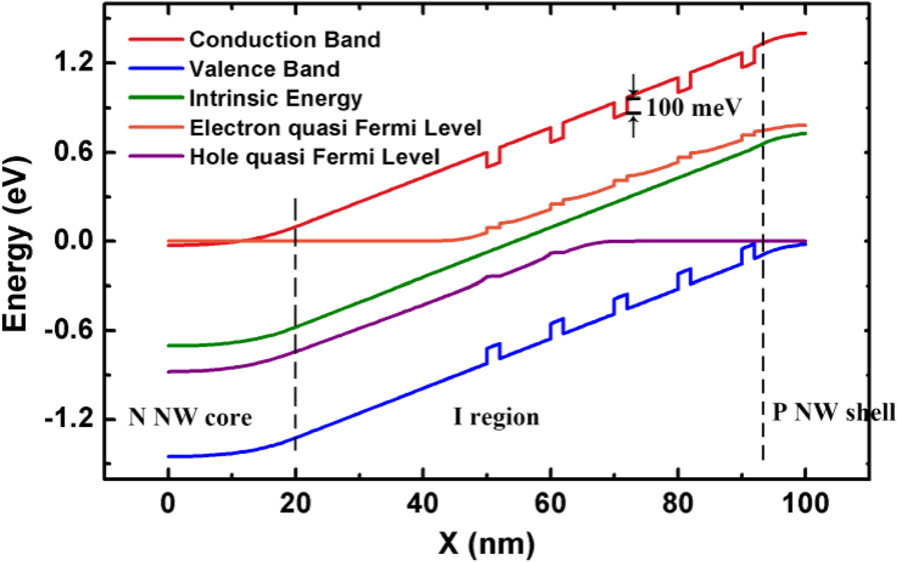
The illuminated energy band diagram of the NW/QD hybrid solar cells

For electrical modeling, the 3D optical generation profiles are incorporated into the finite-element mesh of the devices in the Device software package (Lumerical Solutions, Inc.), which solves the carrier continuity equations coupled with Poisson’s equation self-consistently. To model the carrier transport properties of the effective medium, we assume that the optical generated carriers in GaAs barriers are captured by the lower bandgap 2D WL and, subsequently, relax to the QD ground state on time scales of 1–50 ps [28, 29]. The carriers generated in QDs or captured from WL recombine or escape back to WL through thermal emission [30]. The capture and escape process is modeled by setting 100 meV effective band offsets at the interface of GaAs and effective medium, according to the activation energy for thermal emission from quantum dots reported in literatures [30–32]. A similar modeling method has been reported in [26], in which the characteristics of QD-enhanced multijunction solar cells have been investigated. The illuminated energy band diagram of the NW/QD hybrid solar cells is shown in Fig. 2.

Radiative, Auger, and Shockley-Read-Hall (SRH) recombination is taken into consideration in the device electrical simulation. The QD minority carrier lifetimes are described using a radiative recombination lifetime of 1 ns and a SRH recombination lifetime of 10 ns [26], the final lifetimes of the effective medium are a weighted sum of the QD and GaAs NW recombination lifetimes (assuming carriers captured by the WL are subsequently captured by the QD), as described in [26]. The Auger recombination coefficient of the effective medium is set to 4.2 × 10^−29^ cm^6^/s [33]. And, the electron and hole effective masses are set to 0.053*m*_0_ and 0.341*m*_0_, respectively [26]. In modeling of the transport of barrier carriers across the effective medium region, we use the barrier mobility (2500 cm^2^/Vs for electrons and 150 cm^2^/Vs for holes) [34], as described in [35]. A surface recombination velocity of 3000 cm/s is used in the device model, assuming the nanowire surfaces are well passivated [34, 36]. And, the contact minority carrier recombination velocity is set to be 10^7^ cm/s [37].

## Results and Discussion

**Fig. 3:**
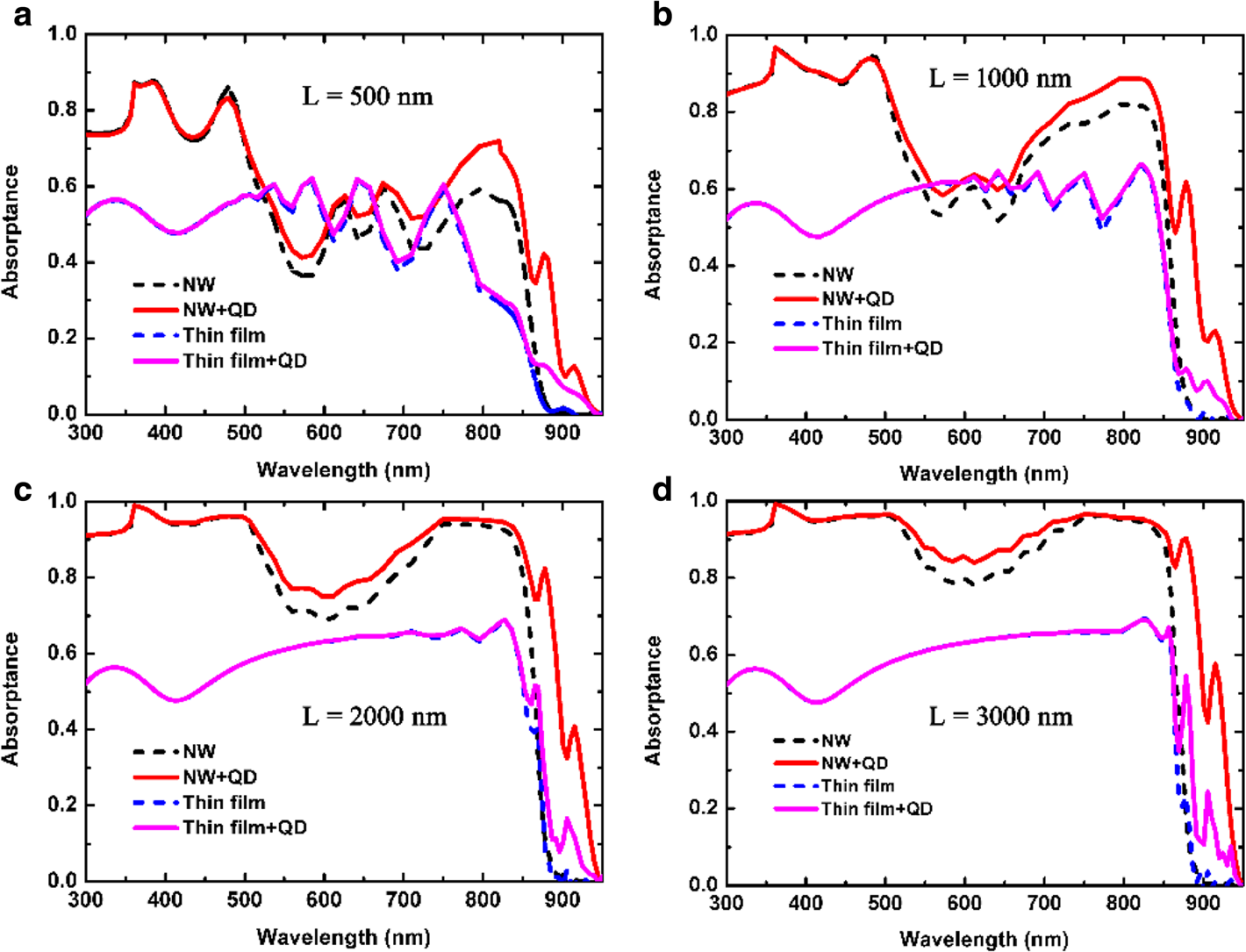
The absorption spectra of the NW/QD hybrid nanostructure array and its thin-film counterpart with and without QD layers. The NW length in **a**–**d** is 500, 1000, 2000, and 3000 nm, respectively

The absorption spectra of GaAs NW array solar cells with and without QD layers are shown in Fig. 3. The NW radius is set to 100 nm, and the period is 360 nm. By introducing QD layers, the absorption of GaAs NWs is dramatically enhanced and the absorption spectrum is extended to 950 nm. Figure 3a–d shows the absorption spectra with different NW lengths. It can be seen that the absorption is markedly increased by QD layers at a wavelength beyond 450 nm, since the QD layers have higher absorption coefficient than GaAs NWs. As the NW length increases, the absorption difference between NW arrays with and without QD layers is getting smaller in the wavelength range beyond the GaAs bandgap, indicating that the absorption of GaAs is more sufficient for longer NWs. While in the wavelength range below the GaAs bandgap, as GaAs NWs contribute little to light absorption, the absorption enhancement induced by QD layers becomes more prominent as the NW length increases. Two absorption peaks are observed in the wavelength range below the GaAs bandgap, which are centered at 876 and 916 nm, respectively, corresponding to the wavelengths at which the effective medium has the highest absorption coefficient. Compared with the NW/QD hybrid solar cell, the absorption of the thin-film solar cell saturates much earlier with the increase of film thickness, as the main loss in the thin-film solar cell is reflection. As the volume ratio of QD layers in the thin films is much lower than that in the NW array, the light absorption enhancement induced by QD layers is almost negligible in the wavelength range beyond the GaAs bandgap. While in the wavelength range below the GaAs bandgap, due to the lack of light-trapping ability, the absorption of QD layers in the thin film is much lower than that in the NW array.

**Fig. 4:**
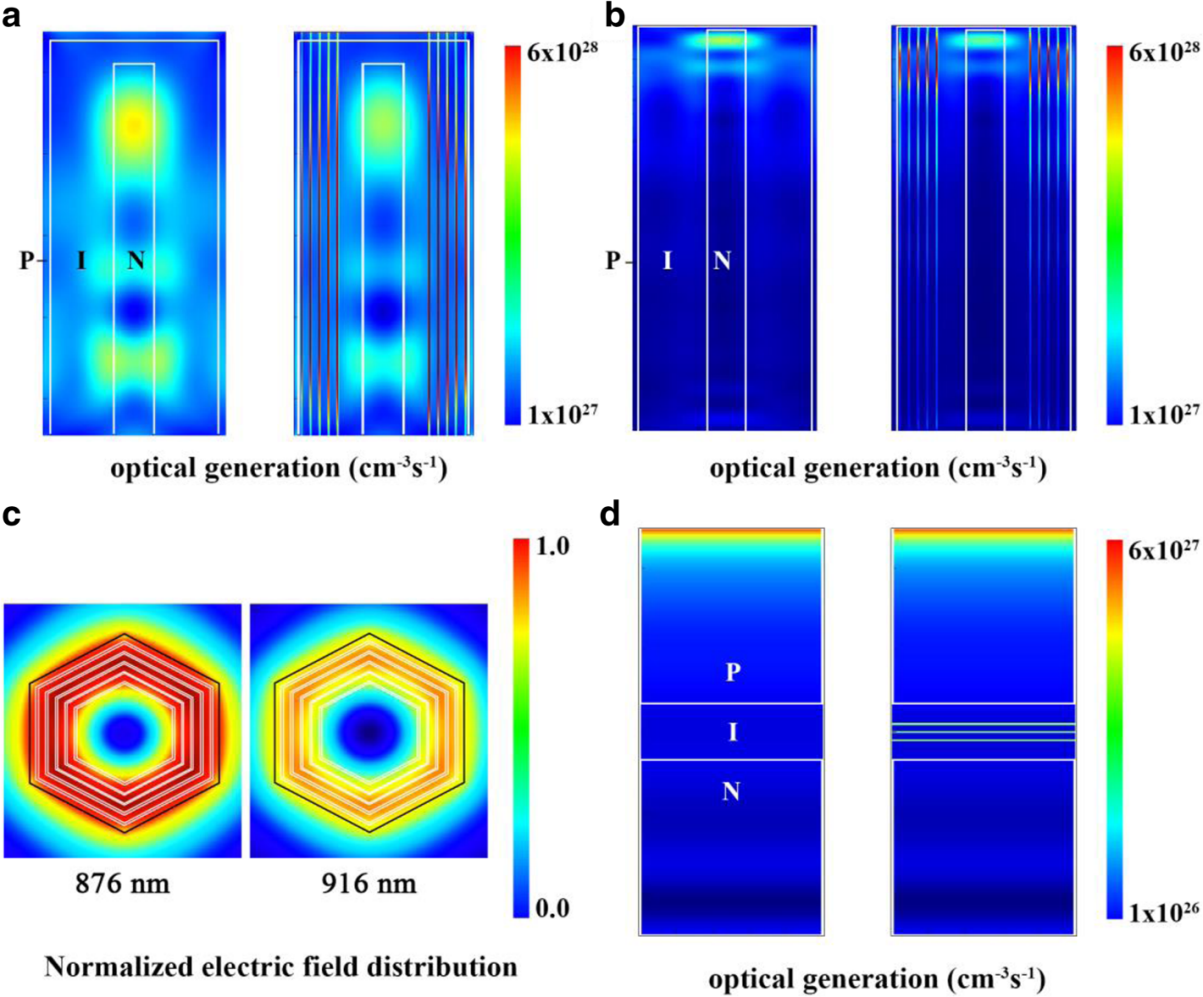
**a** The vertical cross section of optical generation profiles in short NW and NW/QD hybrid solar cells. **b** The vertical cross section of optical generation profiles in long NW and NW/QD hybrid solar cells. **c** The electric field distributions in NW cross section at 876 and 916 nm, in which the location of QD layers is marked out with white lines. **d** The vertical cross section of optical generation profiles in thin-film and thin-film/QD hybrid solar cells

The optical generation profiles of the considered structures are shown in Fig. 4. NWs with lengths of 500 and 3000 nm are considered in this part (hereafter referred to as the short NW and the long NW, respectively). It is obvious that the carrier generation in effective medium is much higher than that in GaAs, demonstrating the absorption enhancement induced by QDs. In NW/QD hybrid solar cells, fewer carriers are generated in the NW core region, since some carriers are concentrated in QD regions instead. This phenomenon is expected to benefit the device performance, as the highly doped core region often suffers from serious recombination loss. In short NWs, the optical generated carriers distribute in the whole NW, while in long NWs, carriers are mainly concentrated at the top, indicating that the light absorption in long NWs is sufficient although the considered NW array does not contain any substrates. It can be observed that, in long NWs, a high carrier generation region in QD layers stretches longer than that in the NW core, and the carriers are concentrated to several lobes along the NW axis. This is induced by the resonance modes at a long-wavelength region in the NW. Long wavelength light has longer absorption length and is mainly absorbed in QD regions, especially the light at a wavelength range below GaAs bandgap. The electric field distributions under unpolarized light illumination in GaAs NWs at 876 and 916 nm are shown in Fig. 4c, from which we can see that the electric field strongly overlaps with the QD regions, which further explains the enhancing effect of NW structures on the QD absorption at this wavelength. The optical generation profile of a 500-nm thin-film solar cell is shown in Fig. 4d, and it can be seen that the absorption in thin-film structures is much weaker than that in NWs. For thin-film structures, the carriers generated in QDs have little effect on the overall generation profile. While in NWs, QDs with the same volume can contribute to absorption significantly thanks to guided-resonance modes in NWs [21].

**Fig. 5:**
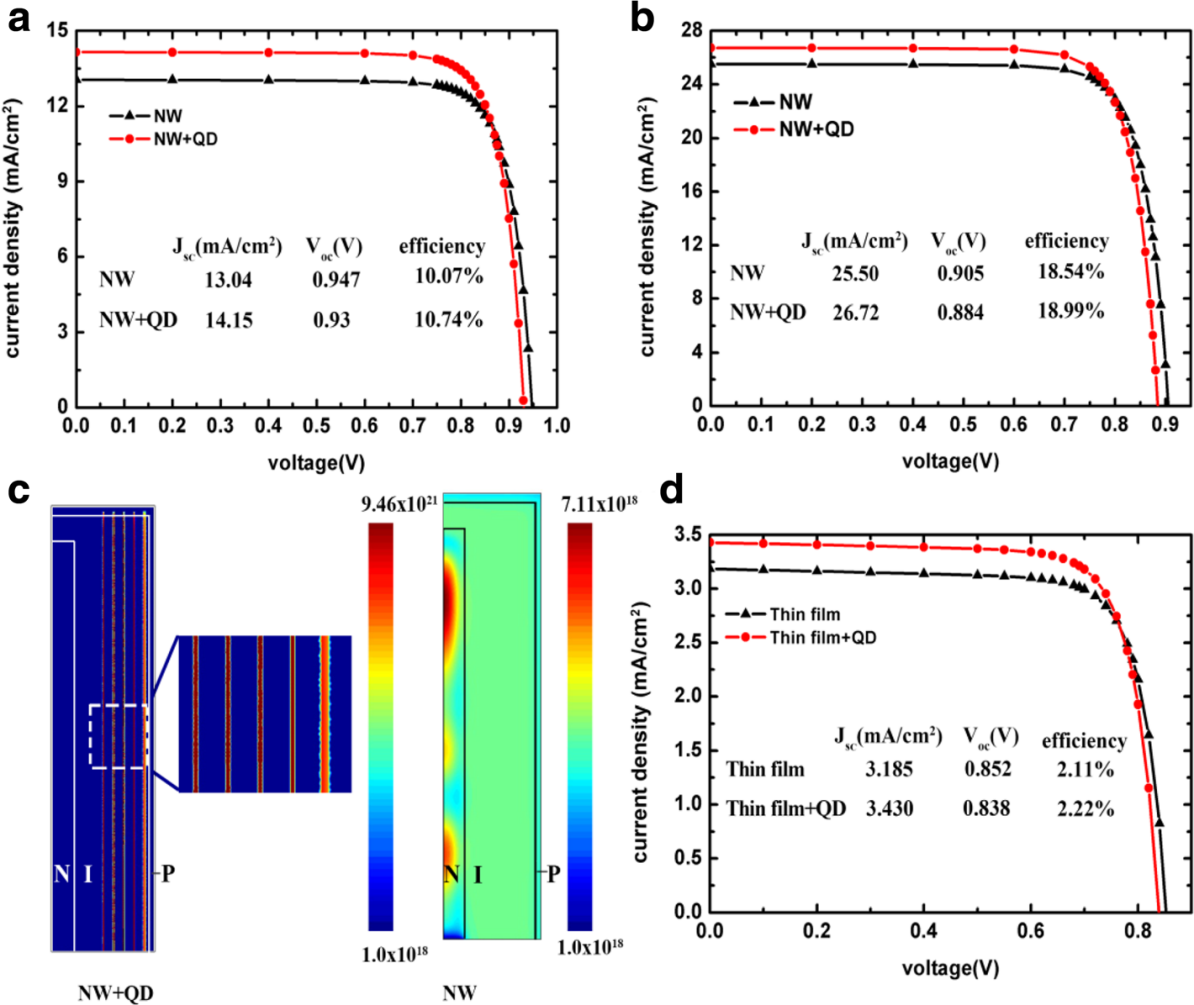
**a** The *I*-*V* characteristics of short NW and NW/QD hybrid solar cells. **b** The *I*-*V* characteristics of long NW and NW/QD hybrid solar cells. **c** Half of the vertical cross section of radiative recombination rates of short NW and NW/QD hybrid solar cells. **d** The *I*-*V* characteristics of thin-film and thin-film/QD hybrid solar cells

Further studies focus on investigation of the potential increase in photovoltaic efficiency gains stemming from the absorption enhancement induced by QDs. Previously simulated photogeneration profiles are incorporated into the Device software package to calculate the *I*-*V* characteristics of considered devices. Carrier generation is expected to be increased in QD regions; however, carriers in QD regions suffer from higher recombination rate. As a result, an enhancement of short-circuit currents (*J*_sc_) in QD-enhanced solar cells is often accompanied by a deterioration of open-circuit voltage (*V*_oc_) [38]. The effect of QDs on device efficiency depends on a trade-off between *J*_sc_ increase and *V*_oc_ reduction. The *I*-*V* characteristics of the NW solar cells are shown in Fig. 5a, b, and corporation of QDs in short NWs leads to a *J*_sc_ enhancement of 1.09 mA/cm^2^ and a *V*_oc_ reduction of 0.017 V. While in long NWs, a *J*_sc_ increase of 1.22 mA/cm^2^ and a *V*_oc_ reduction of 0.021 V are observed. The overall efficiency increase is 0.67% in short NWs and 0.45% in long NWs. By increasing the NW length, the *J*_sc_ enhancement is increased as well as the *V*_oc_ reduction due to the increase of QD volume. Figure 5c illustrates the radiative recombination profiles in NWs near *V*_oc_; compared with pure GaAs NWs, the radiative recombination rates increase by more than 3 orders of magnitude in the QD layers, which explains the *V*_oc_ degeneration. The conversion efficiencies of thin-film solar cells with and without QDs are calculated as well. The efficiency enhancement induced by QDs is only 0.11%, much lower than that in NWs solar cells, although the QD volumes in NWs and thin-film structures are the same. The result indicates that NW array is advantageous to enhancing efficiency of quantum dot solar cells. The efficiency enhancement induced by QDs is not so impressive in this work due to the degeneration of *V*_oc_; however, several approaches have been demonstrated to maintain *V*_oc_ in QD-enhanced solar cells [5, 39]. More satisfying efficiency enhancement is expected to be obtained if the *V*_oc_ degeneration could be avoided in NW/QD hybrid solar cells. Moreover, the optical absorption spectrum of QDs depends strongly on the dot size distribution [40, 41, 42]. We believe that, by modifying the QD size and density, it is possible to achieve higher absorption coefficient, which may lead to more significant absorption enhancement and higher conversion efficiency.

## Conclusions

In summary, we have studied the photovoltaic performance of a GaAs/InAs NW/QD hybrid solar cell. The results show that the absorption spectra of GaAs NWs can be extended to 950 nm by incorporating multilayer InAs QDs on the NW sidewalls. The absorption of QDs is also dramatically improved due to the light-trapping effect of the NW array. *I*-*V* characteristics show that *J*_sc_ in NW solar cells can be increased due to light absorption enhancement, while *V*_oc_ is degenerated because of more serious recombination induced by QDs. The overall efficiency enhancement induced by QDs in NW solar cells is much higher than that in thin-film solar cells, indicating that the GaAs/InAs NW/QD hybrid structure is promising for QD solar cells.

## Abbreviations

3D-FDTD: Three-dimensional finite-difference time-domain
AsH_3_: Arsine
CCS: Close Coupled Showerhead
*I*-*V*: Current density versus voltage
*J*_sc_: Short-circuit current
MOCVD: Metal organic chemical vapor deposition
NWs: Nanowires
QDs: Quantum dots
S-K: Stranski-Krastanov
SRH: Shockley-Read-Hall
TE: Transverse electric
TM: Transverse magnetic
TMGa: Trimethylgallium
TMIn: Trimethylindium
*V*_oc_: Open-circuit voltage
WLs: Wetting layers

## References

[1] Wu J, Yu P, Susha AS, Sablon KA, Chen H, Zhou Z, Li H, Ji H, Niu X, Govorov AO, Rogach AL, Wang ZM (2015). Broadband efficiency enhancement in quantum dot solar cells coupled with multispiked plasmonic nanostars. Nano Energy.

[2] Zheng Z, Ji H, Yu P, Wang Z (2016). Recent progress towards quantum dot solar cells with enhanced optical absorption. Nanoscale Res Lett.

[3] Driscoll K, Bennett MF, Polly SJ, Forbes DV, Hubbard SM (2014). Effect of quantum dot position and background doping on the performance of quantum dot enhanced GaAs solar cells. Appl Phys Lett.

[4] Guimard D, Morihara R, Bordel D, Tanabe K, Wakayama Y, Nishioka M, Arakawa Y (2010). Fabrication of InAs/GaAs quantum dot solar cells with enhanced photocurrent and without degradation of open circuit voltage. Appl Phys Lett.

[5] Bailey CG, Forbes DV, Raffaelle RP, Hubbard SM (2011). Near 1 V open circuit voltage InAs/GaAs quantum dot solar cells. Appl Phys Lett.

[6] Lin C, Liu W, Shih C (2011). Detailed balance model for intermediate band solar cells with photon conservation. Opt Express.

[7] Lee Y, Yao Y, Tsai M, Liu A, Yang M, Lai J (2013). Current matching using CdSe quantum dots to enhance the power conversion efficiency of InGaP/GaAs/Ge tandem solar cells. Opt Express.

[8] Kerestes C, Polly S, Forbes D, Bailey C, Podell A, Spann J, Patel P, Richards B, Sharps P, Hubbard S (2014). Fabrication and analysis of multijunction solar cells with a quantum dot (In) GaAs junction. Prog Photovolt Res Appl.

[9] Walker AW, Theriault O, Hinzer K (2014). The dependence of multijunction solar cell performance on the number of quantum dot layers. IEEE J Quantum Electron.

[10] Tomić S, Jones STS, Harrison NM (2008). Absorption characteristics of a quantum dot array induced intermediate band: implications for solar cell design. Appl Phys Lett.

[11] Luque A, Martí A (1997). Increasing the efficiency of ideal solar cells by photon induced transitions at intermediate levels. Phys Rev Lett.

[12] Bailey CG, Forbes DV, Polly SJ, Bittner ZS, Dai Y, Mackos C, Raffaelle RP, Hubbard SM (2012). Open-circuit voltage improvement of InAs/GaAs quantum-dot solar cells using reduced InAs coverage. IEEE J Photovoltaics.

[13] Yu P, Wu J, Gao L, Liu H, Wang Z (2017). InGaAs and GaAs quantum dot solar cells grown by droplet epitaxy. Sol Energ Mat Sol C.

[14] Mellor A, Luque A, Tobías I, Martí A (2014). The feasibility of high-efficiency InAs/GaAs quantum dot intermediate band solar cells. Sol Energ Mat Sol Cells.

[15] Yan X, Zhang X, Li J, Cui J, Ren X (2015). Fabrication and optical properties of multishell InAs quantum dots on GaAs nanowires. J Appl Phys.

[16] Uccelli E, Arbiol J, Morante JR, Fontcuberta i Morral A (2010). InAs quantum dot arrays decorating the facets of GaAs nanowires. ACS Nano.

[17] Yan X, Zhang Z, Ren X, Lv X, Li J, Wang Q, Cai S, Huang Y (2012). Formation mechanism and optical properties of InAs quantum dots on the surface of GaAs nanowires. Nano Lett.

[18] Yan X, Zhang X, Ren X, Li J, Lv X, Wang Q, Huang Y (2012). Growth and photoluminescence of InxGa1−xAs quantum dots on the surface of GaAs nanowires by metal organic chemical vapor deposition. Appl Phys Lett.

[19] Yan X, Zhang X, Ren X, Huang H, Guo J, Guo X, Liu M, Wang Q, Cai S, Huang Y (2011). Growth of InAs quantum dots on GaAs nanowires by metal organic chemical vapor deposition. Nano Lett.

[20] Jung J, Guo Z, Jee S, Um H, Park K, Lee J (2010). A strong antireflective solar cell prepared by tapering silicon nanowires. Opt Express.

[21] Wen L, Zhao Z, Li X, Shen Y, Guo H, Wang Y (2011). Theoretical analysis and modeling of light trapping in high efficicency GaAs nanowire array solar cells. Appl Phys Lett.

[22] Huang N, Lin C, Povinelli ML (2012). Broadband absorption of semiconductor nanowire arrays for photovoltaic applications. J Opt.

[23] Garnett E, Yang P (2010). Light trapping in silicon nanowire solar cells. Nano Lett.

[24] Yu P, Wu J, Liu S, Xiong J, Jagadish C, Wang ZM (2016). Design and fabrication of silicon nanowires towards efficient solar cells. Nano Today.

[25] Yan X, Zhang X, Li J, Cui J, Wang Q, Huang Y, Ren X (2013). Growth of InAs quantum dots on Si-based GaAs nanowires by controlling the surface adatom diffusion. J Cryst Growth.

[26] Walker AW, Thériault O, Wheeldon JF, Hinzer K (2013). The effects of absorption and recombination on quantum dot multijunction solar cell efficiency. IEEE J Photovolt.

[27] Palik ED (1985). Handbook of optical constants of solids-I.

[28] Ferreira R, Bastard G (1999). Phonon-assisted capture and intradot Auger relaxation in quantum dots. Appl Phys Lett.

[29] Narvaez GA, Bester G, Zunger A (2006). Carrier relaxation mechanisms in self-assembled (In,Ga)As/GaAs quantum dots: efficient P→S Auger relaxation of electrons. Phys Rev B.

[30] Yang W, Lowe-Webb RR, Lee H, Sercel PC (1997). Effect of carrier emission and retrapping on luminescence time decays in InAs/GaAs quantum dots. Phys Rev B.

[31] Brusaferri L, Sanguinetti S, Grilli E, Guzzi M, Bignazzi A, Bogani F, Carraresi L, Colocci M, Bosacchi A, Frigeri P, Franchi S (1996). Thermally activated carrier transfer and luminescence line shape in self-organized InAs quantum dots. Appl Phys Lett.

[32] Fafard S, Raymond S, Wang G, Leon R, Leonard D, Charbonneau S, Merz JL, Petroff PM, Bowers JE (1996). Temperature effects on the radiative recombination in self-assembled quantum dots. Surf Sci.

[33] Bhattacharya P, Ghosh S, Pradhan S, Singh J, Wu Z, Urayama J, Kim K, Norris TB (2003). Carrier dynamics and high-speed modulation properties of tunnel injection InGaAs-GaAs quantum-dot lasers. IEEE J Quantum Electron.

[34] Huang N, Lin C, Povinelli ML (2012). Limiting efficiencies of tandem solar cells consisting of III-V nanowire arrays on silicon. J Appl Phys.

[35] Ramey SM, Khoie R (2003). Modeling of multiple-quantum-well solar cells including capture, escape, and recombination of photoexcited carriers in quantum wells. IEEE T Electron Dev.

[36] Joyce HJ, Docherty CJ, Gao Q, Tan HH, Jagadish C, Lloyd-Hughes J, Herz LM, Johnston MB (2013). Electronic properties of GaAs, InAs and InP nanowires studied by terahertz spectroscopy. Nanotechnology.

[37] Wang X, Khan MR, Lundstrom M, Bermel P (2014). Performance-limiting factors for GaAs-based single nanowire photovoltaics. Opt Express.

[38] Jolley G, Lu HF, Fu L, Tan HH, Jagadish C (2010). Electron-hole recombination properties of In0.5Ga0.5As/GaAsIn0.5Ga0.5As/GaAs quantum dot solar cells and the influence on the open circuit voltage. Appl Phys Lett.

[39] Lam P, Hatch S, Wu J, Tang M, Dorogan VG, Mazur YI, Salamo GJ, Ramiro I, Seeds A, Liu H (2014). Voltage recovery in charged InAs/GaAs quantum dot solar cells. Nano Energy.

[40] Ferreira DL, Alves JLA (2004). The effects of shape and size nonuniformity on the absorption spectrum of semiconductor quantum dots. Nanotechnology.

[41] Tomić S, Sogabe T, Okada Y (2015). In-plane coupling effect on absorption coefficients of InAs/GaAs quantum dots arrays for intermediate band solar cell. Prog Photovolt Res Appl.

[42] Mellor A, Luque A, Tobías I, Martí A (2013). A numerical study into the influence of quantum dot size on the sub-bandgap interband photocurrent in intermediate band solar cells. AIP Adv.

